# Supplementary material for: Beneficial Metabolic Effects of Chitosan and Chitosan Oligosaccharide on Epididymal WAT Browning and Thermogenesis in Obese Rats
Source: Molecules. 2019 Dec 5;24(24):4455. doi: 10.3390/molecules24244455 (PMC6943480; doi:10.3390/molecules24244455)
Supplement: Supplementary file 1 [file molecules-24-04455-s001.pdf]

Supplementary Table 1. Experimental Materials

Materials	Catalog No.	Company
COST (MW≤1,000 Da),	(degree of deacetylation, ≥90%; lot: 160326C)	Shangdong AK Biotech Co., Ltd. (Qingdao, Shandong, China)
COSM (MW≤3,000 Da)	(degree of deacetylation, ≥90%; lot: 160408C)	Shangdong AK Biotech Co., Ltd. (Qingdao, Shandong, China)
CTS	degree of deacetylation, ≥85%; lot: 171112A	Shangdong AK Biotech Co., Ltd. (Qingdao, Shandong, China)
Orlistat	2016120860088	Zhongshan Wanhan Pharmaceutical Co., Ltd. (Guangzhou, GuangDong, China)

Supplementary Table 1. The formula of the high-fat diet (HFD)

Composition	Content (%)
Basic feed	54
Lard	15
Sucrose	15
Milk powder	4
Peanut	3
Egg yolk powder	5
Salt	2
Sesame oil	1
CaHPO4	0.6
Mountain flour	0.4

Supplementary Table 2. Primer sequences for qRT-PCR

Gene	Primer Sequences
UCP1	Forward: 5′-CCAGGCTTCCAGTACTATTAGG-3′
	Reverse: 5′-GTACTCTTGGACCGTATCGTAG-3′
PGC1α	Forward: 5′-TATTCATTGTTCGATGTGTCGC-3′
	Reverse: 5′-TGTCTGTAGTGGCTTGATTCAT-3′
PRDM16	Forward: 5′-GACGCCATAAGAAGTATGCATG-3′
	Reverse: 5′-GATCACATTTGTACTCACGCTC-3′
p38-MAPK	Forward: 5′-AAGACTTCCCAGCAGTCCTATC-3′
	Reverse: 5′-CTGGAGGATCAGTTGTGTTCAA-3′
TMEM26	Forward: 5′- CCATTACCCAGGGAGGAGCA-3′
	Reverse: 5′-AACAACTTGGTTGCTGGGCA-3′
ATF2	Forward: 5′-GAAGCACCGGAACCAAGAGG-3′
	Reverse: 5′-TCTCTGCACGATGCACACAC-3′
Slc27a1	Forward: 5′-GGCTGTGTATGGAGTGGCTGTG-3′
	Reverse: 5′-GGCAGAAGACGCAGGAAGATGG-3′
β-actin	Forward: 5′-GGCTGTATTCCCCTCCATCG-3′
	Reverse: 5′-CCAGTTGGTAACAATGCCATGT-3′
PKA	Forward: 5′-GGACAAGCAGAAGGTGGTGAAGC-3′
	Reverse: 5′-ACCAGGCACGTACTCCATGACC-3′
Dio2	Forward: 5′-GAAGCACCGGAACCAAGAGG-3′
	Reverse: 5′-TCTCTGCACGATGCACACAC-3′
FTO	Forward: 5′-GACCGTCCTGCGATGATGAAGTG-3′
	Reverse: 5′-CCTGTCCACCAAGTTCTCGTCATG-3′
PPARγ	Forward: 5′-CCATCGAGGACATCCAAGACAACC-3′
	Reverse: 5′-GCTCTGTGACAATCTGCCTGAGG-3′
PPARα	Forward: 5′-GCGTACGGCAATGGCTTTAT-3′
	Reverse: 5′-GAACGGCTTCCTCAGGTTCTT-3′

Supplementary Table 3. Primary antibodies for western blotting

Antibodies	Company	Catalog No.	Dilution
UCP1	abcam	ab10983	1/1000
PRDM16	abcam	ab106410	1 - 2 μg/ml
PGC-1α	abcam	ab54481	1/1000
P38-MAPK	abcam	ab170099	1/1000 - 1/5000
ATF2	abcam	ab47476	1/500 - 1/1000
β-actin	abcam	ab5694	0.5 - 2 μg/ml

Supplementary Table 4. Changes of food intake in SD obese rats during administration (n=10, mean±SD)

Group	food intake（g/week）
NF	167.94±5.97
HF	152.02±6.08
Orlistat	175.88±8.34
COST	140.16±10.54
COSM	160.62±10.61
CTS	147.95±4.93

*$p<0.05$，**$p<0.01$ vs HF.

Supplementary Table 5. Changes of body weight in obese SD rats before and after administration and weight gain in obese SD rats（n=10, means±SD）

Group	Body weight before administration（g）	Body weight after administration（g）	Weight gain（g）
NF	446.83±68.28	530.03±89.79	76..44±23.39*
HF	610.19±48.93	700.84±52.78	102.03±26.45
COST	562.73±41.21	610.06±48.97	52.78±20.21**
COSM	573.56±48.2	636.02±65.55	55.78±22.19**
CTS	555.67±31.73	601.9±56.37	60.04±24.75**
Orlistat	546.82±28.17	618.98±36	69.07±20.88*

*p<0.05，**p<0.01 vs HF.

Supplementary Table 6. Fat pad and fat/body ratio (fat content) of obese SD rats in each group（n=10, means±SD）

Group	Epididymal fat (g)	Perirenal fat (g)	Subcutaneous fat (g)	Brown fat (g)	Fat pad (g)	Fat Content (%)
NF	6.47±0.68**	7.52±1.03**	7.21±1.39**	0.52±0.05	20.66±5.93**	4.07±1.07**
HF	15.11±2.65	23.65±3.12	13.99±1.54	0.52±0.08	50.31±3.39	6.47±2.14
COST	9.25±2.7**	14.19±4.4**	12.54±6.08**	0.88±0.11**	31.53±10.81**	5.22±1.42**
COSM	10.48±2.99*	14.32±4.58*	9.79±3.16*	0.84±0.62*	31.59±8.87*	5.04±1.3*
CTS	9.9±2.84**	16.19±6.09**	11.56±5.7**	0.90±0.22**	31.87±10.98**	5.49±1.10**
Orlistat	10.41±1.55**	16.06±0.85**	10.66±1.60**	0.59±0.05	32.08±13.67*	5.11±2*

*p<0.05，**p<0.01 vs HF.

Supplementary Table 7. Serum T-CHO, TG, HDL-C, LDL-C, FFA and Glucose levels in SD rats（n=10, means±SD）

Group	TC (mmol/L)	TG (mmol/L)	HDL-C (mmol/ L)	LDL-C (mmol/L)	FFA (umol/L)	Glucose (umol/ L)
NF	1.55±0.21**	0.53±0.15**	0.32±0.09**	0.42±0.08**	363.24±113.03**	10.83±0.42**
HF	3.25±1.00	1.60±0.65	0.03±0.02	1.46±0.34	611.79±151.83	13.55±0.74
Orlistat	2.25±0.25**	0.75±0.05**	0.16±0.03**	0.60±0.24**	302.85±89.62**	8.12±0.63**
COSM	1.74±0.45**	0.65±0.22**	0.12±0.04**	0.90±0.26**	386.39±145.91*	10.49±0.49**
COST	1.95±0.53**	0.65±0.18**	0.14±0.02**	0.93±0.29**	371.95±180.39**	10.51±0.53**
CTS	2.27±0.17**	0.72±0.19**	0.09±0.05*	1.09±0.24*	373.43±107.15**	9.38±0.57**

*p<0.05，**p<0.01 vs HF.

Supplementary Table 8. Comparison of valid data and reference genome for each group

Sample	Total Mapping (%)	Uniquely Mapping (%)
HF1	93.55	75.83
HF2	92.67	75.1
HF3	93.4	74.32
NF1	93.94	76.11
NF2	93.57	75.75
NF3	93.53	75.18
COSM1	93.58	75.88
COSM2	93.16	73.53
COSM3	93.79	76.96
COST1	93.77	75.77
COST2	93.89	76.73
COST3	93.47	74.86
CTS1	93.53	74.61
CTS2	93.74	76.31
CTS3	94.1	77.61

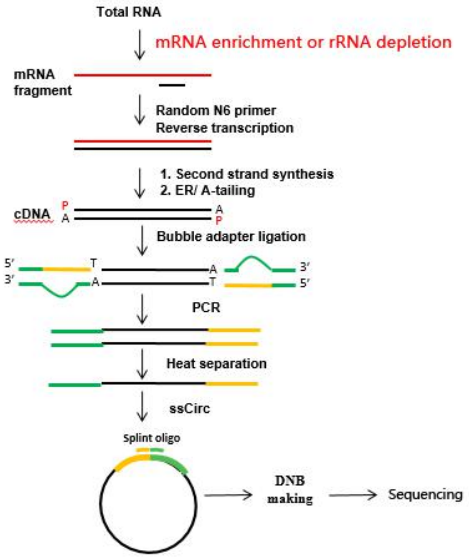

Supplementary Figure 1. mRNA library construction flow chart. Total RNA was extracted from WAT using a commercial kit, and the concentration and quality were detected. The BGI group (Shenzhen, GuangDong) conducted digital gene expression tag profiling. RNA was reverse transcribed to obtain double-stranded cDNA. The PCR product was denatured using heat, and the single-stranded DNA was cyclized using a splint oligo and DNA ligase. The prepared library was sequenced, and subsequently, the differentially expressed genes (DEGs) were identified using the NOISeq method. Then, the DEGs were clustered with Gene Ontology (GO) analysis.

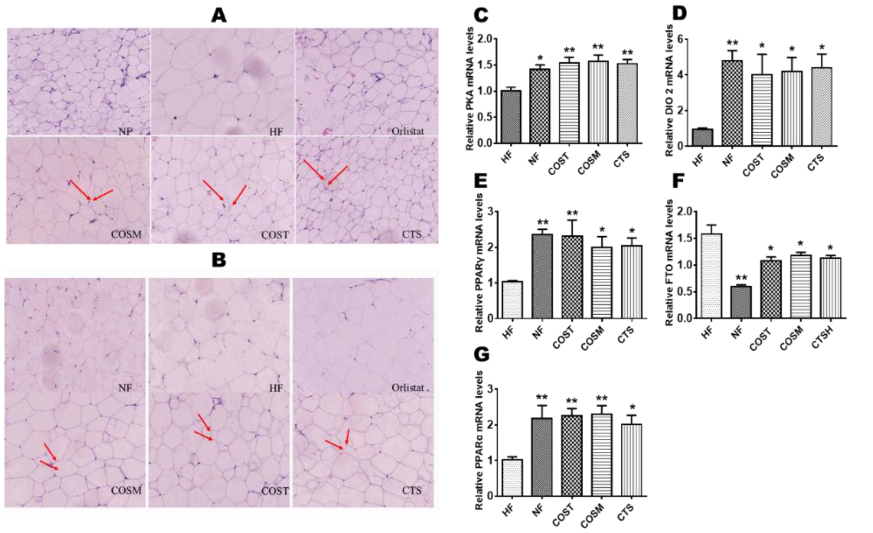

Supplementary Figure 2. CTS, COST and COSM inhibit WAT proliferation, increase liver fatty acid metabolism and promote browning of WAT. A: Histopathology of subcutaneous fat (200×).B: Histopathology of perirenal fat (200 ×). Relative epididymal WAT mRNA expression levels of PKA (C), Dio2 (D), PPARγ (E), and FTO (F) were quantified by qRT-PCR. Relative liver mRNA expression levels of PPARα (G) was quantified by qRT-PCR. Data are expressed as mean ± SD. *P < 0.05 and **P < 0.01vs. HF group.

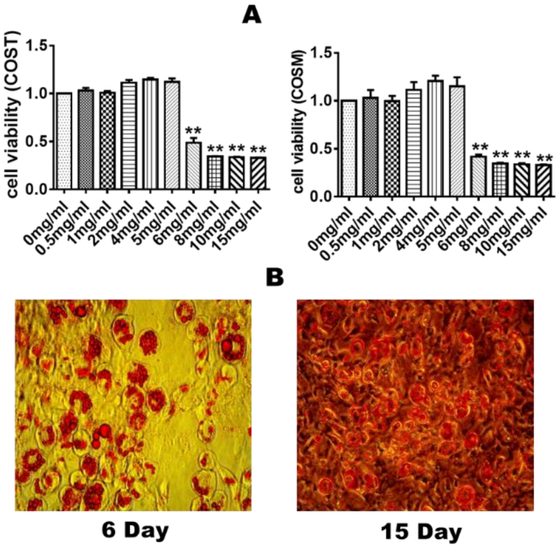

Supplementary Figure 3. Screening of COST and COSM dosing doses and exploration of the time of 3T3-L1 modeling. The CCK8 cytotoxicity test results showed that COST and COSM were added to the 3T3-L1 cell culture medium for 24 h, and the cell viability of the concentration below 5 mg/ml was higher than 90%. Therefore, this experiment selected COST, COSM high, medium and low doses for subsequent experiments, the concentrations were: 0.5mg / ml, 2.5mg / ml, 5mg / ml, recorded as COSMH, COSMM, COSML and COSTH, COSTM, COSTL (A). The results of the oil red O staining experiment showed that after 6 days of induction, the cells became large and saturated, and the cells began to produce ring-shaped lipid droplets. After 15 days of induction, more than 90% of the cells differentiated into mature adipocytes, suggesting that the induction of 3T3-L1 precursor adipocytes to adipocytes was successful (B). Data are expressed as mean ± SD. *P < 0.05 and **P < 0.01 vs. 0mg/mL group.
